# Targeting PIK3CA Actionable Mutations in the Circulome: A Proof of Concept in Metastatic Breast Cancer

**DOI:** 10.3390/ijms23116320

**Published:** 2022-06-05

**Authors:** Barbara Cardinali, Giuseppa De Luca, Roberta Tasso, Simona Coco, Anna Garuti, Giulia Buzzatti, Andrea Sciutto, Luca Arecco, Federico Villa, Franca Carli, Daniele Reverberi, Rodolfo Quarto, Mariella Dono, Lucia Del Mastro

**Affiliations:** 1 Clinica di Oncologia Medica, IRCCS Ospedale Policlinico San Martino, 10-16132 Genova, Italy; andrea.sciutto@hsanmartino.it (A.S.); arecco.luca@gmail.com (L.A.); lucia.delmastro@hsanmartino.it (L.D.M.); 2 Molecular Diagnostic Unit, IRCCS Ospedale Policlinico San Martino, 10-16132 Genova, Italy; maria.dono@hsanmartino.it; 3 Department of Experimental Medicine (DIMES), University of Genova, 5-16126 Genova, Italy; roberta.tasso@unige.it (R.T.); rodolfo.quarto@unige.it (R.Q.); 4 Lung Cancer Unit, IRCCS Ospedale Policlinico San Martino, 10-16132 Genova, Italy; simona.coco@hsanmartino.it; 5 Clinica Oncologia Medica ad Indirizzo Oncologico, IRCCS Ospedale Policlinico San Martino, 10-16132 Genova, Italy; anna.garuti@hsanmartino.it; 6 Oncologia Medica 2, IRCCS Ospedale Policlinico San Martino, 10-16132 Genova, Italy; giulia.buzzatti@hsanmartino.it; 7 Molecular Oncology and Angiogenesis Unit, IRCCS Ospedale Policlinico San Martino, 10-16132 Genova, Italy; vilfed@libero.it; 8 Surgical Pathology Unit, IRCCS Ospedale Policlinico San Martino, 10-16132 Genova, Italy; franca.carli@hsanmartino.it; 9 Molecular Pathology Unit, IRCCS Ospedale Policlinico San Martino, 10-16132 Genova, Italy; daniele.reverberi@hsanmartino.it; 10 Cellular Oncology Unit, IRCCS Ospedale Policlinico San Martino, 10-16132 Genova, Italy; 11 Department of Internal Medicine and Medical Specialties (DIMI), University of Genova, 5-16126 Genova, Italy

**Keywords:** breast cancer, *PIK3CA* mutations, liquid biopsy, circulating tumor DNA, circulating tumor cells, extracellular vesicles

## Abstract

The study of circulating cancer-derived components (circulome) is considered the new frontier of liquid biopsy. Despite the recognized role of circulome biomarkers, their comparative molecular profiling is not yet routine. In advanced breast cancer (BC), approximately 40% of hormone-receptor-positive, HER2-negative BC cases harbor druggable *PIK3CA* mutations suitable for combined alpelisib/fulvestrant treatment. This pilot study investigates *PIK3CA* mutations in circulating tumor DNA (ctDNA), tumor cells (CTCs), and extracellular vesicles (EVs) with the aim of determining which information on molecular targetable profiling could be recollected in each of them. The in-depth molecular analysis of four BC patients demonstrated, as a proof-of-concept study, that it is possible to retrieve mutational information in the three components. Patient-specific *PIK3CA* mutations were found in both tissue and ctDNA and in 3/4 cases, as well as in CTCs, in the classical population (large-sized CD45−/EpCAM+/− cells), and/or in the “non-conventional” sub-population (smaller-sized CD44+/EpCAM−/CD45− cells). Consistent mutational profiles of EVs with CTCs suggest that they may have been released by CTCs. This preliminary evidence on the molecular content of the different circulating biomaterials suggests their possible function as a mirror of the intrinsic heterogeneity of BC. Moreover, this study demonstrates, through mutational assessment, the tumor origin of the different CTC sub-populations sustaining the translational value of the circulome for a more comprehensive picture of the disease.

## 1. Introduction

In oncology, circulome refers to whole cancer-derived components released by the primary tumor and/or metastatic sites into the bloodstream, including circulating tumor (Ct)-derived DNA/RNA, circulating tumor cells (CTCs), and extracellular vesicles (EVs) [1,2,3]. Much evidence has been developed on the clinical application of both ctDNA and CTCs [4]. Although the use of EVs in clinical practice is still in its first steps and needs more investigation, these biological particles are new, promising tools for cancer pantients’ management [5].

CtDNA comprises short nucleic fragments (80–200 bp) released into circulation by apoptosis and/or necrosis and provides mainly quantitative and qualitative information through mutational profiling by allowing the identification of genetic alterations associated with treatment susceptibility and response, hence supporting decision making for personalized management [6]. In BC, as well in lung cancer, ctDNA analysis has already entered the clinic to guide treatment decisions [7,8].

CTCs are a rare and highly heterogeneous circulating population, released into the bloodstream by the primary tumor or secondary lesions. New advanced technologies have allowed their detection and characterization, demonstrating the potential relevance of their enumeration as a prognostic predictor for many cancers, including metastatic breast, colon and lung cancers [9,10,11,12,13]. However, the implementation of their analysis in the clinic is still a matter of debate since both the counting and characterization of CTCs are far from being completely robust and established processes, mainly because their detection is based only on their EpCAM expression or on their size [14]. In this regard, the analysis of other CTC sub-populations, which have lost their epithelial features and have already been identified in different tumors [15] as “non-conventional” CTCs [16], is becoming of primary interest.

EVs are heterogeneous, lipid-bound vesicles secreted by all cell types into the extracellular space [17]. The three main sub-types of EVs are exosomes, microvesicles, and apoptotic bodies, which are differentiated based upon their biogenesis, release pathways, size, content, and function [18]. Since EVs carry complex molecular cargoes, such as proteins, RNA, and DNA fragments, they can be considered reliable fingerprints of parental cells, delivering potentially useful information for cancer prognosis/progression [19,20,21,22,23].

Breast cancer (BC) is the most common cancer in women and is characterized by highly heterogeneous traits with various genomic characteristics and clinical behaviors that pose great challenges in achieving disease control and care. In the BC clinical context, the analysis of circulating tumor-derived materials is emerging as a novel approach for patient management in advanced and early BC settings [6,7]. The main investigations of circulome analytes in BC have focused primarily on the quantitative and qualitative aspects of ctDNA and CTCs [24,25,26]. In fact, high levels of ctDNA have been associated with a more aggressive and potentially resistant disease, both in early and advanced settings. In addition, the identification of mutations in plasma-derived ctDNA can predict some clinical behavior such as human epidermal growth factor receptor 2 (*HER2)* amplification in the onset of anti-HER2 therapy resistance [27] or estrogen receptor 1 (*ESR1)* gene mutation in the development of aromatase inhibitor treatment resistance [28]. Among the various circulating biomarkers, CTC count in BC plays a well-defined and specific clinical role [29,30,31], being an independent factor of disease-free survival (DFS) and overall survival (OS) in both early and metastatic disease. Identification of ≥5 CTCs/7.5 mL of blood is a strong predictor of a worse outcome in metastatic BC patients, regardless of other parameters such as imaging tools or serum antigens [32,33,34,35]. Growing evidence shows that EVs from BC cells contribute to carcinogenesis and may serve as a good candidate for cancer diagnosis (as biomarkers), as well as therapy (as drug carriers) [36,37]. In BC, most of the data acquired on the different circulating biomarkers are derived from studies conducted in the BC metastatic setting, where specific mutations, detectable by liquid biopsy, have already been proven to be the cause of drug resistance or susceptibility to target treatment. For patients with hormone receptor (HR)-positive, HER2-negative metastatic BC, standard treatment is based on endocrine therapy, although tumors frequently develop resistance, primarily due to the presence of activating mutations in phosphatidylinositol-4,5-bisphosphate 3-kinase (*PIK3CA*; 40% of cases) [38]. These *PIK3CA*-mutated BCs have been shown to be susceptible to therapy with alpelisib in preclinical tumor models [39] and clinical trials in association with fulvestrant [40,41]. For treatment decision making, in 2019, the FDA approved the detection of *PIK3CA* mutations in ctDNA on the bases of a companion test, confirming the use of liquid biopsy in the BC clinical context. Despite great advances in circulome analysis, comparative studies on the molecular profiling of ctDNA, CTCs, and EVs have not yet been routinely performed. With this in mind, we investigated whether *PIK3CA* mutations in the BC metastatic setting could be identified among the three different circulome components. Since this is a proof-of-concept study, we took advantage of a small cohort of patients with advanced HR+/HER2- BC who were candidates for therapy with alpelisib and performed analyses to determine which information on the targetable molecular profile could be collected from the different biological analytes also in comparison with those present in the corresponding tumor tissue.

## 2. Results

**Figure 1:**
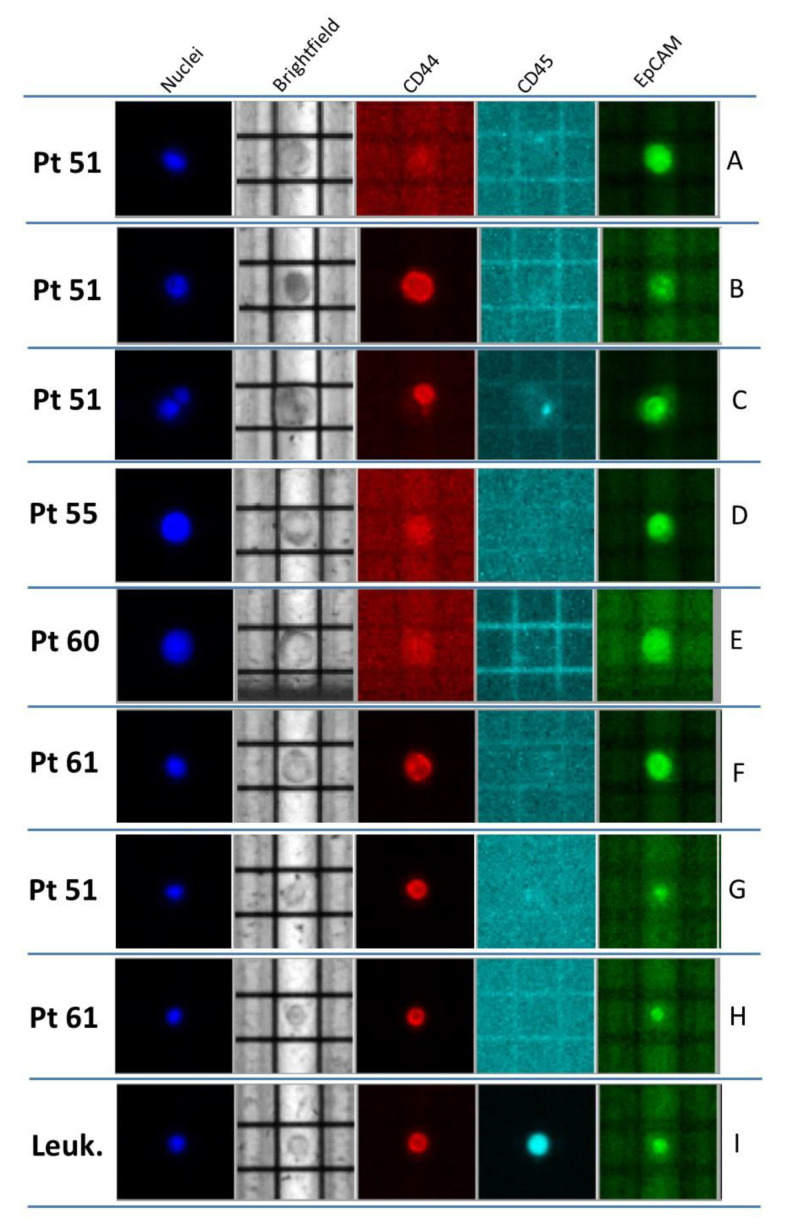
Representative CTCs recovered from the four BC patients enrolled. (**A**–**F**) Classical CTCs, identified as round, middle-sized cells; CD45-negative, EpCAM-/CD44-positive or -negative, and Hoechst-positive cells. (**A**,**D**) EpCAM-positive CTCs; (**B**,**F**) CD44-positive CTCs; (**C**) heterogeneous CTC cluster (composed of one EpCAM-positive cell and one CD44-positive cell); (**E**) EpCAM- and CD44-negative CTC. (**G**,**H**) “Non-conventional” CTCs, identified as small-sized CD45-negative and CD44-positive cells; (**I**) example of leukocyte (CD45-positive and, very often, CD44-positive cells).

**Figure 2:**
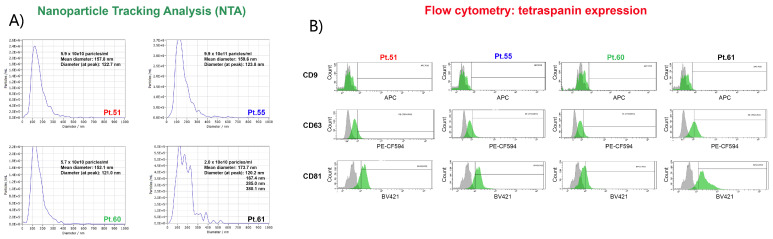
(**A**) EV NTA analysis shows very similar profiles in terms of EV populations detected in all patient samples but reveals a very peculiar nanoparticle distribution for Pt# 61, which may deserve further investigation. (**B**) Expression of typical EV markers CD9, CD63, and CD81 evaluated by flow cytometry on a BD FACS Aria. (**Upper panel**) The expression of CD9 was almost absent in EVs consistently in all four patients; (**middle panel**) CD63 was detected in a comparable percentage in Pt#51, Pt#55, and Pt#60, while for Pt#61, it shows an almost double expression; (**lower panel**) EVs presented a similar expression of CD81 in Pt#51, Pt#55, and Pt#61 samples, while in Pt#60, it presented a lower expression. Some like 2.0E+5 mean 2.0 × 10^5^.

Four patients were enrolled in this study. All patients were diagnosed as HR+/HER2- BC and had metastatic disease at the time of enrollment. Clinical characteristics and therapy regimens are reported in Table 1. All of them were found to be *PIK3CA* mutated at the time of progression; each patient’s specific mutations were followed in the different circulating biomarkers in comparison with the corresponding tumor tissue (details in Table 2 and Table 3, Figure 1 and Figure 2).

### 2.1. Case 51

Patient 51, 40 years old at diagnosis, was treated in 2013 with radical mastectomy and axillary node dissection for locally advanced ductal breast cancer (pT2 pN3a). She received adjuvant chemotherapy, followed by radiotherapy and adjuvant hormone therapy. Four years from diagnosis, the patient experienced bone relapse and was treated with first-line therapy with CDK4/6 inhibitors plus aromatase inhibitors.

The *PIK3CA* p.(Hys1047Arg) mutation was identified in tissue, and the patient received alpelisib and fulvestrant as second-line therapy. ctDNA analysis confirmed the *PIK3CA* alteration at a frequency of 2.3%**.**

A total of 41 CTCs was detected in blood, and almost half (21/41) were negative with respect to the selected markers; 17 of them showed epithelial origin (EpCAM), and only 3 CTCs expressed CD44. Two different pools of “conventional” CTCs containing three and six cells were mutationally analyzed. In both pools, the *PIK3CA* p.(Hys1047Arg) originally present in tumor tissue and ctDNA were detected. Of note, this patient also presented clusters comprising either only CTCs (an example is reported in Figure 1C) or CTCs together with WBC. As previously shown, the presence of these cell clusters is considered a negative prognostic factor [42].

The concentration of EVs was 5.9 × 10^10^ particles/mL. Nanoparticle Tracking Analysis (NTA) revealed that the mean EV diameter was 157.8 nm with a peak at 122.7 nm, indicating that the majority of isolated EVs were composed of exosomes (Figure 2A). CD81 was the only tetraspanin highly expressed (82.4%) by EVs. Indeed, only 13.2% of EVs expressed CD63, while CD9 was absent from these EVs (Figure 2B). Notably, the *PIK3CA* patient-specific variant was also detected in the EV-DNA (1.4%) by droplet digital pPCR (ddPCR).

### 2.2. Case 55

Patient 55 was diagnosed in 2004 at the age of 43 years old with locally advanced HR+ ductal BC and clinical-stage cT4b N1. She received neoadjuvant and adjuvant chemotherapy and subsequent adjuvant radiotherapy and hormone therapy. Eight years later, bone recurrences were found. At further progression of bone disease, she was tested for *PIK3CA* (p.(Glu542Lys), 14.3%) in tissue.

**Figure 3:**
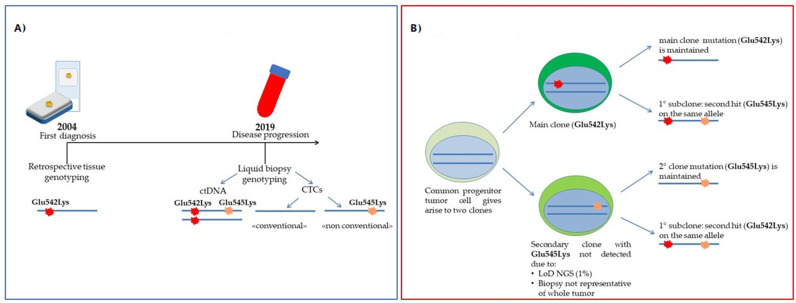
Case of Pt#55. (**A**) Timeline of mutational assessment; (**B**) Hypothesis of the clonal evolution of the disease: the different mutations detected in CTCs may reflect tumor heterogeneity.

The next-generation sequencing (NGS) test on ctDNA revealed two *PIK3CA* mutations: the first confirmed that already found in tissue, p.(Glu542Lys) at 7.5%, and the second, p.(Glu545Lys) (4%), was expressed in perfect cis configuration with the first p.(Glu542Lys) and never represented as a single alteration (Figure 3).

A total of 18 CTCs was detected in the blood; only 5 expressed EpCAM and none CD44. Six pools of “classical” CTCs were investigated for molecular characterization, but none of them showed the *PIK3CA* p.(Glu542Lys) mutation, the primary *PIK3CA* mutation found as a hallmark of tumor tissue. Surprisingly, one pool of 10 “non-conventional” cells showed the *PIK3CA* p.(Glu545Lys) variation alone and without other co-occurring mutations.

Interestingly, the concentration of EVs in this patient was particularly high (9.9 × 10^11^ particles/mL), with a mean diameter of 159.6 nm and a peak at 123.8 nm (Figure 2A). Moreover, in this case, CD81 was the most expressed tetraspanin (82.2%), while 17% of EVs were CD63 positive, and CD9 was absent (Figure 2B). Surprisingly, in EV-DNA, we identified only the *PIK3CA* Glu545Lys mutation, indicating a mutational status similar to that of non-conventional CTCs.

### 2.3. Case 60

Patient 60, 65 years old at diagnosis, was found in 2018 to have de novo metastatic breast cancer (multiple bone metastasis). She received first-line therapy with CDK4/6 inhibitors and aromatase inhibitors. Following bone disease progression in 2019, according to the presence of *PIK3CA* mutation, she was treated with alpelisib and fulvestrant.

In the tumor tissue, the mutational analysis revealed a p.(Hys1047Leu) *PIK3CA* mutation at 36.9% frequency, and the ctDNA NGS analysis validated the tissue mutation but also identified a second mutation in the *ESR1* gene (p.(Asp538Gly), 0.39%), usually associated with endocrine resistance.

In this patient, a total of 19 CTCs was detected. Differently from the other cases, the majority of them (16/19) had a strong epithelial origin since they expressed the EpCAM marker, none showed the CD44 antigen, and a minority of CTCs (3/19) were negative for both these markers (EpCAM-/CD44-). Four pools of “conventional” CTCs (range: 2–3 CTCs per pool) were analyzed. None of them showed *PIK3CA* p.(Hys1047Leu), thus not recapitulating the mutational profiles of the tissue or ctDNA. Similar data were also found in two pools of 10 “non-conventional” cells.

The concentration of circulating EVs was 5.7 × 10^10^ particles/mL, with a mean diameter and a peak diameter of 152.1 nm and 121 nm, respectively (Figure 2A). In contrast to the previous patients, Pt#60-EVs were not highly expressing CD81 (34.3%), whereas 20.4% were CD63 positive, and in accordance with the other EV populations here described, CD9 was not expressed (Figure 2B). Similar to CTCs, the EV-DNA also did not harbor *PIK3CA* p.(Hys1047Leu) or other mutations.

### 2.4. Case 61

Patient 61, 41 years old, was diagnosed in 2015 with locally advanced HR+/HER2- BC. She started neoadjuvant chemotherapy, and after surgery, she received adjuvant radiotherapy and hormone therapy. In 2019, bone, node, and liver metastases were found. She received first-line therapy with CDK4/6 inhibitors and fulvestrant, and after the detection of the p.(Glu542Lys) (51.6%) *PIK3CA* mutation by both tissue and ctDNA tests, she received alpelisib and fulvestrant.

In addition, NGS analysis of ctDNA showed the p.(Gly245Ser) mutation at 33.5% variant allele frequency (VAF) in the *TP53* gene, also detectable in tumor tissue (25.5% VAF).

This case showed the lowest number of CTCs detected in our cohort since only eight cells were recovered. These CTCs were identified mostly by morphological parameters since only two cells expressed EpCAM, and one cell expressing the CD44 marker. One pool of five CTCs underwent NGS analysis, confirming the presence of both *PIK3CA* p.(Glu542Lys) and *TP53* p.(Gly245Ser) mutations. Furthermore, three pools of 10 “non-conventional” CTCs were also tested, and in 1/3 of them, *PIK3CA* p.(Glu542Lys) was detected co-occurring with the *TP53* p.(Gly245Ser).

Despite the concentration of EVs (2 × 10^10^ particles/mL), Pt#61 was in line with the data obtained from Pt#51 and Pt#55 plasma samples, and their size distribution was profoundly different. Indeed, the mean diameter was 173.7 nm, with multiple peaks at 120.2, 167.4, 173.7, 285, and 380 nm, suggesting the presence of a heterogeneous population (Figure 2A). Flow cytometry analysis revealed that EVs highly expressed CD81 (89.5%) and an increased expression of CD63 (47.9%), contrary to the previously described patients. The tetraspanin CD9 was, again, absent, as already discussed for the other EVs patient populations (Figure 2B). In addition, the EV-DNA shared the same *PIK3CA* p.(Glu542Lys) mutation already observed in ctDNA and CTCs.

## 3. Discussion

The characterization of the different circulating biomarkers and the integration with the analyses of their mutational profiles could provide a new, more complete, reliable, and longitudinally repeatable tool in oncology. Different studies have been conducted in this direction, but most of them took into account two of the circulating biomarkers at the time, such as ctDNA and CTCs [43], ctDNA and EVs [44], or the comparison of one circulating biomarker with tissue [45,46]. In this study, we attempted to compare *PIK3CA* mutations in the metastatic BC clinical setting within three components of liquid biopsy, i.e., ctDNA, CTCs including “non-conventional” CTCs, and EVs isolated from four patients.

Comparable results were obtained when tissue and ctDNA (Table 2) were analyzed, providing informative data about *PIK3CA* mutations in all four BC patients. These results are in line with the literature for advanced settings, as this analysis has already been approved in clinics with specific respect to alpelisib treatment [41]. Therefore, the detection of *PIK3CA* mutations in ctDNA is not surprising. However, the case of Pt#55 (Figure 3A,B) pointed out the advantage that the use of ctDNA analysis could add to the standard molecular profiling of tumor tissue since it more specifically details the evolution of the neoplasia. Indeed, the identification of the second mutation in the *PIK3CA* gene, not detected in the primary tumor, could be interpreted as disease progression (Figure 3). Moreover, the presence of mutations in *ESR*1 and *TP53* detected in Pt#60 and Pt#61, respectively, confirms that the ctDNA analysis could provide additional insights into the routine mutational profile of tissue. In this regard, our data further support the approach of longitudinal monitoring through liquid biopsy, already an essential tool in the precision medicine era.

As for CTC analysis (Table 2 and Table 3), some considerations should be made. CTC numbers ≥ 5/7.5 mL, with similar morphological and phenotypic features (Table 3, Figure 1), were evident in all four patients (range: 8–41 cells), as expected in the advanced BC setting. The isolation method and characterization used in this study allowed us to go beyond classical large EpCAM+ CTCs, selecting sub-groups of CTCs with different morphological and phenotypic characteristics. The presence of classical EpCAM+ CTCs was evident in all patient samples, but large-sized EpCAM- CTCs were also detected with different span numbers (range: 5–21). Finally, the CD44 marker was rarely detected among classical CTCs. Even CD44+ cells were identified in two out of four patient samples, and their number was very low (1–2 cell/patient). These data confirm the importance of the analysis of different sub-groups of CTCs, in particular of those cells not expressing epithelial features and defined in the literature as “non-conventional” CTCs [16,47]. In previous studies, the tumor origin of “non-conventional” CTCs has been addressed by copy number variations through whole-genome amplification (WGA). Here, applying our optimized WGA-free NGS workflow to simultaneously test different gene regions [48], we were able to address the tumor origin of these cells. Furthermore, here, we took advantage of marker-independent CTC enrichment and the single-cell recovery DEPArray System workflow, which allowed identifying and isolating an additional sub-class of cells in all four patient samples. In details, these cells were smaller than those described above (nuclei radius range: 7–9 µm), morphologically similar to WBC but negative for the CD45 marker, and only expressing CD44 (Figure 1 G,H). Of note, we also demonstrated the tumor origin of these cells thanks to the detection of somatic mutations occurring in the *PIK3CA* and *TP53* genes that were previously identified in tumor tissue (Table 2). All these findings together support the importance of the complete analysis of CTCs, including “non-conventional” CTCs, since, in some cases, they may add more detail to the mutational dynamics and clonal evolution of the tumor. The fact that in Pt#55 the secondary *PIK3CA* p.(Glu545Lys) (only found in ctDNA but not in primary tumor) was found only in “non-conventional” CTCs, leads to the hypothesis of a model of clonal evolution where, from a common tumoral progenitor, two different cells arose (Figure 3 A,B). The main clone would develop *PIK3CA* p.(Glu542Lys), populating the primary tumor. This clone may develop on the same allele the secondary *PIK3CA* p.(Glu545Lys) alteration under some pressure; a second minor clone, arising from the common unmutated progenitor, would harbor only the secondary *PIK3CA* p.(Glu545Lys), and this would probably be the original cause of “non-conventional” CTCs. These findings confirm the high heterogeneity of BC and the challenge of its therapeutic intervention [49]. Moreover, these data, once again, evidence the importance of including molecular data from circulating biomarkers in the whole clinical assessment for more precise patient management.

When EVs were analyzed, we observed that the average concentration and size distribution were quite similar across the patients (Table 3), except for Pt#61, whose EVs showed a higher average size (173.7 nm vs. 156.5 nm) and a more heterogeneous profile characterized by multiple peaks (Figure 2A). This patient showed the highest mutational frequency (around 50% in *PIK3CA* and 30% in *TP53*), which may reflect the disease dynamic. Analysis of the tetraspanin family member expression (Figure 2B) was similar among patients, and the CD9 antigen was negative, but CD81 and CD63 were present even with different extents and denoted a variability in line with recent literature [50]. Although the DNA copy number from EVs was lower compared to ctDNA results, we confirmed the *PIK3CA* mutation in EV-DNA, as already reported [51]. In addition, we showed that the mutational EV-DNA profiles were consistent with the CTC results (both classical and “non-conventional” CTCs). For example, in Pt#55, the p.(Glu545Lys) mutation standing alone in EV-DNA and “non-conventional” CTCs leads us to speculate that CTCs could be the direct source of circulating mutated EVs. The latter data fit well with the results of different studies and were confirmed by our personal experimental experience, supporting the findings that EV content in oncologic patients is higher compared to healthy controls [52] and that their concentration could be related to CTCs and, therefore, to the potential metastatic power of the disease.

In the BC clinical context analyzed in this study, our results confirmed that the mutational status of ctDNA always resembles the tissue characteristics and pointed out that circulating biomarkers reveal additional information (e.g., novel *TP53* and *ESR1* alterations in Pt#61 and Pt#60). These findings may add important mutational details in the course of the disease and further help to better tailor the therapy. Among the different components of the circulome, in our hands, ctDNA performed the best. This is not surprising considering that some technical standardization of the ctDNA extraction method and NGS analysis procedure have been already reached, and although ctDNA testing is not yet a complete substitute to tissue genotyping, it has already been implemented in the clinic for various tumors, including BC for addressing the *PIK3CA* mutation. All the above data support the idea that an integrated analysis that includes both CTCs and EVs has potential clinical value since they may represent more suitable and complementary tools for studying the clonal evolution of the tumor. We are aware that, at this time, the inclusion of CTC and EV analyses into routine clinical workflows can be technically challenging; however, this opportunity could indeed be very important for a highly selected cohort of BC patients, such as women with multi-organ metastases or hardly accessible secondary tumor sites, such as Patient 55 in this study.

We are also conscious that the results of this pilot study need to be validated in a wider cohort of BC patients. However, despite the exiguous number of cases analyzed, our study represents a contribution to the knowledge of the biological dynamics of *PIK3CA* in the circulome.

## 4. Materials and Methods

### 4.1. Patient Selection

With the goal of assessing the biological characteristics and molecular profiles of the circulome biomarkers, a homogeneous cohort of patients was chosen. Therefore, as a proof of concept, four metastatic HR+/HER2- BC patients, with known *PIK3CA* mutations as candidates to be treated with alpelisib, were selected from those enrolled in a translational trial conducted at IRCCS Ospedale Policlinico San Martino (CITUCEL protocol approved by Regione Liguria Ethics Committee, 4 February 2016, Operative Decision No. 0519, 4 May 2016). All patients provided written informed consent before enrollment.

### 4.2. Tissue Mutational Analysis: Custom-Designed NGS Panel Testing

As previously described [48], we developed an NGS panel in order to perform molecular profiling of the most frequent DNA somatic mutations in BC. Briefly, the panel, based on AmpliSeq technology, included 291 amplicons within 20 genes (i.e., *TP53, PIK3CA, ERBB2, ERBB3, ERBB4, ESR1, MCL1, GATA3, PTEN, CCND1, KRAS, AKT1, CDH1, MAP2K4, SF3B1, FBXW7, MAP3K1, PIK3R1, EGFR*, and *FGFR1*). Where necessary, tissue macro-dissection was applied to enrich the neoplastic cells (>50%). A total of 10–15 ng was addressed to DNA library preparation, and subsequent sequencing was performed in Ion Torrent GeneStudio^TM^ S5 (Thermo Fisher Scientific, Waltham, USA). The output BAM and VCF files were analyzed in Ion Torrent Suite and Ion Reporter v. 5.16 (Thermo Fisher Scientific) and further displayed in Integrative Genomic Viewer (IGV, Broad Institute, Cambridge, MA, USA).

### 4.3. Circulome Processing Workflow

**Figure 4:**
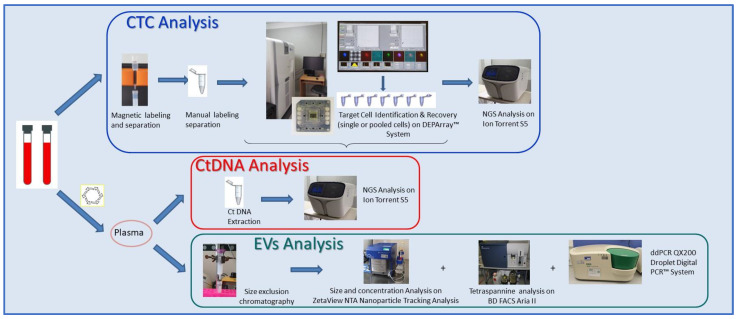
Schematic representation of the parallel analytical workflows performed for ctDNA, CTC, and EV analyses.

For each patient, a peripheral blood sample (20 mL) was collected in an EDTA tubes before treatment with alpelisib and processed within 2 hours according to the workflow reported in Figure 4. The three different circulome components were retrieved from blood or plasma and further characterized with specific protocols.

### 4.4. Isolation, Extraction, and Quantification of ctDNA

Plasma was isolated from blood by two different centrifugation steps (4 °C, 10 min each): the first at 1600 g and the second at 16,000 rpm. The resulting plasma was stored at –80 °C until DNA extraction. ctDNA was extracted from 3 to 5 mL of plasma using the Maxwell^®^ RSC cfDNA Plasma Kit (Promega, Milan, Italy) for the large-volume protocol, according to the manufacturer’s instructions. Isolated ctDNA was then quantified by Qubit™ using the dsDNA HS Assay Kit (Thermo Fisher Scientific).

### 4.5. CTC Selection, Recovery, and Characterization

Cell recovery and selection from 7.5 mL of whole blood were performed, as previously reported [48,49]. Briefly, after red blood cell lysis and immunomagnetic negative selection on LD separation columns (Miltenyi Biotec, Bologna, Italy) using antibodies coupled to magnetic beads against common leukocytes and red blood cell antigens (CD45 and CD235 MicroBeads, Miltenyi Biotec), cells were labeled with anti-EpCAM fluorescein isothiocyanate (FITC) conjugate (Clone 9C4, BioLegend, Koblenz, Germany), anti-CD44 phycoerythrin (PE) conjugate (Clone 51, BD Biosciences, Milano, Italy), and anti-CD45 allophycocyanin (APC) conjugate (Clone 5B1, Miltenyi Biotec) antibodies and Hoechst (Merck Life Science, Milano, Italy), then fixed and permeabilized for complete nuclei staining with the Fix and Perm kit (Miltenyi Biotec). Cell enumeration and sorting were performed by the DEPArray^TM^ System (Menarini-Silicon Biosystems, Bologna, Italy), following the manufacturer’s standard procedure. “Classical” CTC selection and characterization were performed based on standard morphological and phenotypic criteria [48]: (i) round or oval shape; (ii) presence of a clear Hoechst-stained nucleus; (iii) high nuclear–cytoplasmic rate; (iv) CD45 negativity; (v) eventual EpCAM and/or CD44 positivity. A sub-population of CD45−/CD44+ cells, with different features from the “classical” CTCs, i.e., smaller size cell, 7–9 µm average nuclei, and morphologically similar to leukocytes, was detected and named as “non-conventional” CTC population. Pools of 2–10 cells from both classical and/or “non-conventional” CTCs were recovered. Finally, CD45+ small-sized leukocytes were isolated and used as negative controls. CTC enumeration was performed by two independent investigators.

### 4.6. EV Isolation and Characterization

EVs were isolated from 900 µL of plasma by size-exclusion chromatography (SEC) (qEV Original/70 nm; Izon¸ Christchurch, New Zeland), according to the manufacturer’s instructions. Then, all the collected fractions were analyzed, and their protein elution profiles were obtained by monitoring the absorbance at a wavelength of 280 nm. A 10 µL volume of EV-enriched pooled fractions was used to evaluate EV size and concentration by nanoparticle tracking analysis (NTA) using the ZetaView NTA (Particle Matrix, Ammersee, Germany). EV characterization by flow cytometry was performed, as previously described [50,53]. For each preparation, one tube was stained with 1 μM of CFDA-SE at 4 °C (Vybrant™ CFDA-SE Cell Tracer Kit, Thermo Fisher Scientific) as a control to verify CFDA-SE specificity, and one tube containing EVs was stained with the same amount of CFDA-SE at RT to visualize intact EVs and set the correct dimensional gate. A mixture of fluorescent beads of varying diameters (Megamix-Plus FSC and Megamix-Plus SSC, Biocytex, Marseille, France) was used following the manufacturer’s instructions to discriminate EV size. Expression of typical EV markers CD9 (312108, APC Mouse Anti-Human CD9, BioLegend), CD63 (565403, PE-CF594 Mouse Anti-Human CD63, BD Horizon), CD81 (740079, BV421 Mouse Anti-Human CD81, BD Biosciences), and the corresponding isotype controls APC Mouse IgG1, κ Isotype Ctrl (FC) Antibody (Clone MOPC-21, Biolegend), PE-CF594 Mouse IgG1, κ Isotype Ctrl (FC) Antibody (562292, BD Horizon), and BV421 Mouse IgG1, κ Isotype Ctrl Antibody (562438, BD Biosciences) were evaluated within the CFDA-SE-positive events using BD FACS Aria II (BD Biosciences). EV samples also underwent DNA extraction using the QIAamp DNA Micro Kit (Qiagen, Milan, Italy), following the manufacturer’s instructions.

### 4.7. ctDNA Mutational Analysis

NGS Oncomine™ Breast v2 cfDNA Assays (Thermo Fisher Scientific) were used to detect ctDNA mutations in a total of 152 hotspots, including copy number variation within 13 genes (i.e., *AKT1, EGFR, ERBB2, ERBB3, ESR1, FBXW7, KRAS, PIK3CA, SF3B1, CCND1, ERBB2, FGFR1*, and *TP53*). Library quantification and size evaluation were checked using TapeStation 2200 and High Sensitivity D1000 ScreenTape (Agilent Technologies, Santa Clara, CA, USA). Each barcoded library was diluted to 50 pM and used for template preparation in the Ion Chef System and by using Ion 540™ chips (Thermo Fisher Scientific). Sequencing (coverage: ≥25,000×) was performed in Ion Torrent GeneStudio^TM^ S5, as indicated by the manufacturer’s specifications.

### 4.8. CTC Whole-Genome Amplification-Free Library Preparation and Sequencing

The DNA libraries of isolated CTC pools were prepared by using the Optimized Molecular Tagging NGS Workflow as already reported [48] and opportunely designed to skip the WGA step. Briefly, CTC pool lysates were used as starting nucleic material and processed with Oncomine Breast cfDNA Research Assay v2 (Thermo Fisher Scientific). Libraries were quantified and multiplexed up to 24 different barcoded samples on an Ion 520™ chip, and low-coverage sequencing was achieved. NGS runs and analysis were performed in Ion Torrent GeneStudio™ S5 and Ion Torrent Suite, respectively. Reads were then visualized in Integrative Genome Viewer to check the presence of variation.

### 4.9. PIK3CA Status by Droplet Digital PCR (ddPCR)

The mutational status of *PIK3CA* on circulome molecules, including ctDNA and EV-DNA, was investigated by the QX200 Droplet Digital PCR™ System (Bio-Rad Laboratories, Inc., Hercules, CA, USA) using PIK3CA mutation probe-based assays (c.1624G > A, p.(Glu542Lys); c.1633G > A, p.(Glu545Lys); c.3140A-T, p.(His1047Leu); c.3140A-G, p.(His1047Arg)). For each reaction, 5–10 µL of ctDNA or EV-DNA was amplified, as already reported [54]. In each run, 2 to 4 sample replicates were run with a wild-type sample, positive DNA (DNA from PIK3CA+/cell lines), and no template control. Then, data were analyzed using QuantaSoft software v. 1.7.4.0917 (Bio-Rad Laboratories) in 2D amplitude, and mutation positivity was defined by applying channel thresholds based on no pattern, wild-type DNA signals, and positive control. The allele fraction was calculated as merged replicates, and the positivity was called when at least 2 *PIK3CA* mutant droplets were detected.

## 5. Conclusions

To the best of our knowledge, this is the first study that analyzed the *PIK3CA* mutational status within different circulating components, providing new insight into the use of liquid biopsy.

First, our evidence shows that it is possible to recover, at the same time, mutational information from ctDNA, CTCs, and EVs. Second, the experimental data point out that while ctDNA represents the most sensible and easiest biomarker to obtain, CTCs could be an additional biological source to be used for the characterization of tumor heterogeneity. In addition, EV phenotype, size distribution, and mutational status might be the mirror of evidence of the disease. Third, this study demonstrates the tumor origin of the different CTC sub-populations (including “non-conventional” CTCs) through mutational assessment. The analysis of these cell sub-types, if confirmed in a larger population, may be included in routine BC liquid biopsy, to draw a more complete picture of the disease.

## Figures and Tables

**Table 1:** Patient characteristics: tumor biological features and therapy regimens.

Pt	Age at First Diagnosis	Biopsy and staging	NAC	Surgery and Restaging	AC, RT, HT	DFS	Site of 1st Recurrence	1st-Line Therapy	PFS I	2nd-Line Therapy	PFS II
**PT#51**	2013 40 years	/	No	Ductal BC pT2, G2 pN3a ER: 40% PgR: 40% Ki67:8%	EC (4 cycles) + T (12 cycles) + RT + LHRHa and Tam	4 ys	Bones	CDK4/6 Inhibitors + AI	22 ms	Alpelisib + fulvestrant	NA
**Pt#55**	2004 43 years	Ductal BC cT4b cN1 ER: 75% PgR: 5% Ki67: 24%	E + C + D (6 cycles)	Ductal BC ypT1c, G2 pN1a ER: 15% PgR: 0% Ki67: 25%	C + M + F (3 cycles) + RT + Tam for 2 ys, then AI for 3 ys	8 ys	Bones	CDK4/6 Inhibitors + AI	12 ms	Alpelisib + fulvestrant	7 ms
**Pt#60**	2018 65 years	Ductal BC M1 ER: 90% PgR: 70%Ki67: 20%	No	/	/	/	Bones	CDK4/6 Inhibitors + AI	21 ms	Alpelisib + fulvestrant	7 ms
**Pt#61**	2015 41 years	Ductal BC ER: 96% PgR: 86% Ki67: 18%	EC (4 cycles) + T (12 cycles)	Ductal BC ypT1c, G2 pN1a ER: 93% PgR: 16% KI67: 6%	RT + LHRHa and AI	4 ys	Bones, nodes, liver	CDK4/6 Inhibitors + fulvestrant	5 ms	Alpelisib + fulvestrant	3 ms

Legend: ER: estrogen receptor; PgR: progesterone receptor; NAC: neoadjuvant therapy; AC: adjuvant chemotherapy, RT: radiotherapy; HT: hormone therapy; DFS: disease-free survival; PSF: progression-free survival; EC: epirubicin and cyclophosphamide; T: paclitaxel; C: capecitabine; D: docetaxel; Tam: tamoxifen; M: methotrexate; F,: fluorouracil; ms: months; ys: years; AI: aromatase inhibitors; LHRHa: luteinizing hormone-releasing hormone analog; NA: not available.

**Table 2:** Mutational status of the different circulating components in comparison with the corresponding tumor tissue.

Pt ID	Tissue	CTCs		ctDNA		EVs
	Mutational Status by NGS	Conventional CTCs	Non-Conventional CTCs	Mutational Status by NGS	*PIK3CA* Status by ddPCR	*PIK3CA* Status by ddPCR
**#51**	*PIK3CA*, Hys1047Arg	*PIK3CA*, Hys1047Arg	not tested	*PIK3CA*, Hys1047Arg (2.3%)	*PIK3CA*, Hys1047Arg (2.2%)	*PIK3CA*, Hys1047Arg (1.4%)
**#55**	*PIK3CA*, Glu542Lys	WT	*PIK3CA*, Glu545Lys	*PIK3CA*, Glu542Lys (7.5%) Glu545Lys (4.0%)	*PIK3CA*, Glu542Lys (8.1 %) Glu545Lys (4.0%)	*PIK3CA*, Glu545Lys *
**#60**	*PIK3CA*, Hys1047Leu	WT	WT	*PIK3CA*, Hys1047Leu (0.7%); *ESR1*, Asp538Gly (0.39%)	*PIK3CA*, Hys1047Leu (0.7%)	WT
**#61**	*PIK3CA*, Glu542Lys; *TP53*, Gly245Ser	*PIK3CA*, Glu542Lys; *TP53*, Gly245Ser	*PIK3CA*, Glu542Lys; *TP53*, Gly245Ser	*PIK3CA*, Glu542Lys (51.6%); *TP53*, Gly245Ser (33.5%)	*PIK3CA*, Glu542Lys (48.8%)	*PIK3CA* Glu542Lys (54.6%)

* In this case, the ddPCR identified just two positive droplets out of 20 wild-type (WT) *PIK3CA* droplets.

**Table 3:** CTC and EV physical and phenotypic properties.

Pt ID	Conventional CTCs				EVs				
	Number	EpCAM−	EpCAM+	CD44	Number	Size	CD9	CD81	CD63
#51	41	21	17	3	5.90 × 10^10^	157.8	Neg	82.4%	13.2%
#55	18	13	5	0	9.90 × 10^11^	159.6	Neg	82.2%	17.0%
#60	19	3	16	0	5.70 × 10^10^	152.1	Neg	34.3%	20.4%
#61	8	5	2	1	2.00 × 10^10^	173.7	Neg	89.5%	47.90%

## Data Availability

NGS data are available at: https://dataview.ncbi.nlm.nih.gov/object/PRJNA823520?reviewer=dhiuomt13gtr1aqkjl4ls9rfdf (accessed on 5 April 2022).
